# Association between Age at Menarche and Hypertension among Females in Southern China: A Cross-Sectional Study

**DOI:** 10.1155/2019/9473182

**Published:** 2019-11-03

**Authors:** Wei Zhou, Tao Wang, Lingjuan Zhu, Minghua Wen, Lihua Hu, Xiao Huang, Chunjiao You, Juxiang Li, Yanqing Wu, Qinghua Wu, Huihui Bao, Xiaoshu Cheng

**Affiliations:** ^1^Center for Prevention and Treatment of Cardiovascular Diseases, The Second Affiliated Hospital of Nanchang University, Nanchang, Jiangxi, China; ^2^Department of Cardiovascular Medicine, The Second Affiliated Hospital of Nanchang University, Nanchang, Jiangxi, China

## Abstract

**Background:**

Association between age at menarche (AAM) and hypertension remains a controversial topic, and data in China were sparse. Therefore, we aimed to investigate the association between AAM and hypertension in Chinese female population.

**Methods:**

In this cross-sectional study, 5,102 females aged ≥15 years were enrolled. Self-reported AAM was assessed by the questionnaire. Multiple linear regression analysis was used to evaluate the association between systolic blood pressure (SBP), diastolic blood pressure (DBP), and AAM. Logistic regression analysis was performed to evaluate the association between hypertension and AAM. Generalized additive model (GAM) and smooth curve fitting (penalized spline method) were conducted to explore the exact shape of curve between them.

**Results:**

The overall mean of AAM was 15.5 years. Each additional year of AAM was associated with a 15% higher risk of hypertension (odds ratio (OR) = 1.15, 95% confidence interval (CI): 1.11–1.19). Among females with hypertension, there was a significant positive association between AAM and SBP (*β* = 0.88, 95% CI: 0.29–1.46) and DBP (*β* = 0.80, 95% CI: 0.47–1.13). A significantly higher risk of hypertension was found in younger subjects (15–44 y: OR = 1.37, 95% CI: 1.21–1.55; *P* for interaction = 0.009) compared with those aged between 62 and 97 y.

**Conclusions:**

AAM was positively associated with hypertension and blood pressure, especially among females in early adulthood from southern China.

## 1. Introduction

AAM is the onset of the first menstrual cycle, which represents the beginning of the capacity to reproduce, and can influence the health in later life of females [1]. Previous researches have shown that an early AAM was linked to increase the risk of type 2 diabetes [2, 3], obesity [4, 5], metabolic syndrome [6], cardiovascular diseases (CVD), and all-cause mortality in the later adult life [7]. Although growing evidence has established the association of AAM with hypertension, conflicting results could be found among those reports. Canoy et al. [7] reported that there was a U-shaped pattern of relationship between AAM and hypertension in UK female population. For females with AAM up to 13 years, the risk of hypertension reduced as AAM increased, whereas, for those with AAM over 13 years, the risk of hypertension increased with AAM. Compared with AAM at 13 years, AAM at ≤10 and ≥17 years was significantly associated with increased relative risks (and 95% CIs) of 1.14 (1.12–1.16) and 1.08 (1.04–1.11) for hypertension. Previous studies found that an early AAM was related to higher blood pressure (BP) and hypertension, in China, Korea, Singapore, or western countries [8–11]. However, Liu et al. [12] revealed that females with late menarche had a higher risk of hypertension in southwestern China. Moreover, the nonlinearity of AAM and hypertension was rarely analyzed. We hypothesized that the association between AAM and hypertension might vary among ethnic groups, and there might be a nonlinear relationship between them. Therefore, we investigated AAM and hypertension in Chinese females.

## 2. Methods

### 2.1. Ethics Statement

Ethical approval was obtained from the ethics review boards of the Second Affiliated Hospital of Nanchang University and the Fuwai Cardiovascular Hospital (Beijing, China). All participants who wrote informed consent were enrolled in the study. If the participants were unable to write, fingerprinting was used. The ethics committee approved the procedure.

### 2.2. Study Population

**Figure 1:**
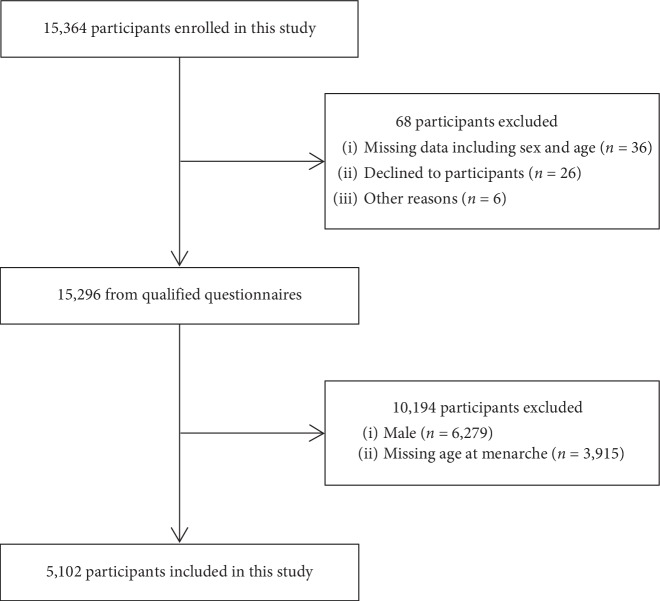
Flow chart of study participants.

We followed the methods of Yu et al. [13], and all investigators were not aware of the hypothesis. Using a stratified multistage random sampling method, a total of 15,364 eligible participants aged ≥15 years were enrolled in our study, and 15,296 of them completed the assessment from November 2013 to August 2014. After excluding males (*n*=6279) and participants with missing data for AAM (*n*=3880) and BP/hypertension (*n*=35), a total of 5102 participants were included in the final analysis (Figure 1).

### 2.3. Questionnaire Survey

Participants were required to finish a standardized questionnaire which was developed by the national coordinating center of Fuwai Cardiovascular Hospital (Beijing, China) through face-to-face interviews. The questionnaire included demographic information (such as age, gender, residence marital status, and education), behavioral characteristics (including smoking, drinking, physical activity, and sleeping duration), and medical history (hypertension, MI, and stroke, which were collected and verified with medical or hospital records). The survey staff received training on data collection before the survey.

### 2.4. Physical Examination

Weight was measured using an Omron body fat and weight measurement device (V-BODY HBF-371; Omron, Kyoto, Japan) to the nearest 0.01 kg in light clothing without shoes indoors. Height was measured without shoes using a standard right-angle device and a fixed vertical ruler to the nearest 0.1 cm. Waist circumference (WC) was obtained by a measuring tape to the nearest 0.1 cm as the minimum circumference at the midpoint between the costal margin and iliac crests (at the level of umbilicus) in light clothing. Body fat percentage (BFP), visceral adiposity index (VAI), and basal metabolic rate (BMR) were also measured using an Omron body fat and weight measurement device (V-BODY HBF-371; Omron, Kyoto, Japan). All measurements were taken twice, and the average of the 2 values was used. Systolic BP (SBP), diastolic BP (DBP), and heart rate (HR) were measured 3 times on the right arm positioned at the heart level with an electronic BP monitor (Omron HBP-1300; OMRON, Kyoto, Japan) after a rest for 5 minutes, with a 30-second interval between each measurement, and the average value of 3 readings was used for analysis [14]. Participants were asked to avoid vigorous exercise, smoking, drinking, and consumption of coffee and tea for at least 30 minutes before the measurements.

### 2.5. Definitions

AAM was defined as the age in whole years at the first menstrual period. Age at menopause was the age in whole years at the last menstrual period, whether natural or resulting from hysterectomy or ovariectomy. Hypertension was defined as previously diagnosed hypertension with or without current treatment or the average SBP ≥ 140 mmHg and/or DBP ≥ 90 mmHg at physical examinations [15, 16]. Body mass index (BMI) was calculated as the weight (kg)/height (m^2^) and classified into groups of <18.5, 18.5–23.9, 24.0–27.9, and ≥28.0 kg/m^2^. Education level was divided into 3 domains according to the number of years of education (0 to 6, 7 to 9, and ≥10 years). Alcohol drinking was defined as drinking alcohol at least one time per week during the previous year. Smoking was defined as never, current (daily smoking, >6 months), and former (cessation of smoking, >6 months) [17–19]. Physical activity was assessed and classified by the International Physical Activity Questionnaire (IPAQ), which was supported by the WHO and the Centers for Disease Control (CDC) [20, 21]. Each individual was grouped as performing low, moderate, or vigorous physical activity, according to the questionnaire scoring protocol (standard scoring criteria at http://www.ipaq.ki.se). Sleep duration was based on the response to the questions: “How many hours of sleep do you get in a 24-hour period on a weekday and nonworkday, respectively? ” The weighted average of the weekly sleep duration was calculated by using the formula: (sleep duration on weekdays ×5 + sleep duration on the weekend ×2)/7 [22].

### 2.6. Statistical Analysis

Continuous variables were presented as the means (standard deviation, SD) or medians (interquartile range), and categorical variables were expressed as percentages among the overall subjects and by AAM stratified into ≤13, 14-15, 16-17, and ≥18 years. Continuous variables were checked for normality and were logtransformed, if necessary. Categorical variables were analyzed using the chi-square test, and continuous variables were analyzed by one-way analysis of variance (ANOVA). Multiple linear regression analysis was carried out to evaluate *β* and 95% CIs between SBP, DBP, and AAM. Multivariate binary logistic regression was performed to evaluate the odds ratio (ORs) and 95% CIs of hypertension being associated with AAM. We built three sets of models based on clinical experiences and literatures. Crude model controlled for none. Model 1 controlled for age, residence, education, and marital status. Model 2 controlled for all variables in model 2 plus smoking, drinking, menopause, antihypertensive medication, physical activity, BMI, WC, BFP, VAI, BMR, HR, and sleeping duration. Multicollinearity was checked among all covariates included in model 2, and none of variables violated the criteria. Subgroup analysis by age, education, marital status, smoking, drinking, menopause, physical activity, and BMI was conducted to test for interactions between AAM and the potential confounders by including cross-product interaction terms in the corresponding multiple linear regression models. Moreover, to explore the nonlinear relationship between AAM and hypertension, a generalized additive model (GAM) and smooth curve fitting (penalized spline method) were conducted. All tests of statistical significance were based on *P* < 0.05. All analyses were done with Empower (R) (https://www.empowerstats.com; X&Y Solutions, Inc, Boston, MA), and the forest plot was generated by R software, version 3.1.2 (http://www.r-project.org).

## 3. Results

### 3.1. Characteristics of Subjects

As shown in Table 1, a total of 5102 females (aged from 15 to 97 years, with a mean of 52.6 years at enrollment) were included in this study. The mean of AAM (aged from 9 to 24 years) for the total sample was 15.5 years. The overall prevalence of hypertension was 29.3% in our study. Compared with females with early AAM, females with a later AAM were more likely to have a larger mean enrollment age, BMI, WC, and also a higher BFP, VAI, SBP, DBP, prevalence of hypertension, rate of antihypertension medication, and menopause, and tended to engage in more vigorous physical activity (all *P* < 0.05). Females with an earlier AAM were tended to be unmarried and engaged in more low or moderate physical activity. In addition, 2700 out of 5102 were postmenopausal females, and their mean ages at menopause (SD) across AAM categories (≤13, 14-15, 16-17, and ≥18 years) were, respectively, 48.0 (6.3), 47.9 (5.1), 48.5 (6.8), and 48.2 (5.5) with no significant difference (*P*=0.860).

### 3.2. Association between AAM and Hypertension

**Figure 2:**
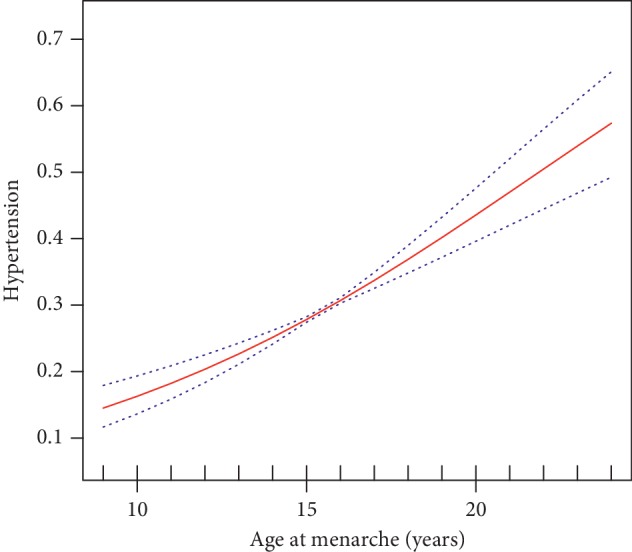
Multivariate adjusted smooth curve of hypertension by AAM. The solid line represents the estimated values, and the dashed line represents the 95% CI. Adjusted for enrollment age, residence, education, marital status, smoking, drinking, menopause, antihypertensive medication, physical activity, BMI, WC, BFP, VAI, BMR, HR, and sleeping duration.

Table 2 presents OR and 95% CI for hypertension according to AAM. After adjusting all confounders, per 1 year increase of AAM was associated with 15% increased risk of hypertension (*P* < 0.001). Compared with AAM ≤13 year, ORs (95% CIs) for hypertension across AAM at 14-15, 16-17, and ≥18 years were 0.95(0.76, 1.19), 1.68(1.34, 2.11), and 2.01(1.56, 2.58), respectively (*P* for trend <0.001). However, there was no significant difference between females with AAM at ≤13 years and 14 to 15 years, so we combined the two AAM groups in Table 2. Compared with females with AAM at ≤15 years, the fully adjusted ORs (95% CIs) for hypertension across AAM at 16-17 and ≥18 years were, respectively, 1.74 (1.48, 2.05) and 2.08 (1.71, 2.53). In the present study, the adjusted smooth curve showed the linear association between AAM and hypertension (Figure 2).

### 3.3. Association between AAM and SBP, DBP

Among hypertensive females, AAM was significantly positively correlated with SBP (*β* (95% CI) = 0.88 (0.29, 1.46)) and DBP (*β* (95% CI) = 0.80 (0.47, 1.13)) (Table 3). When AAM was divided into ≤15, 16-17, and ≥18 years, each stratum of AAM was positively associated with SBP and DBP (*P* for trend <0.001).

### 3.4. Hypertension and AAM by Subgroups

**Figure 3:**
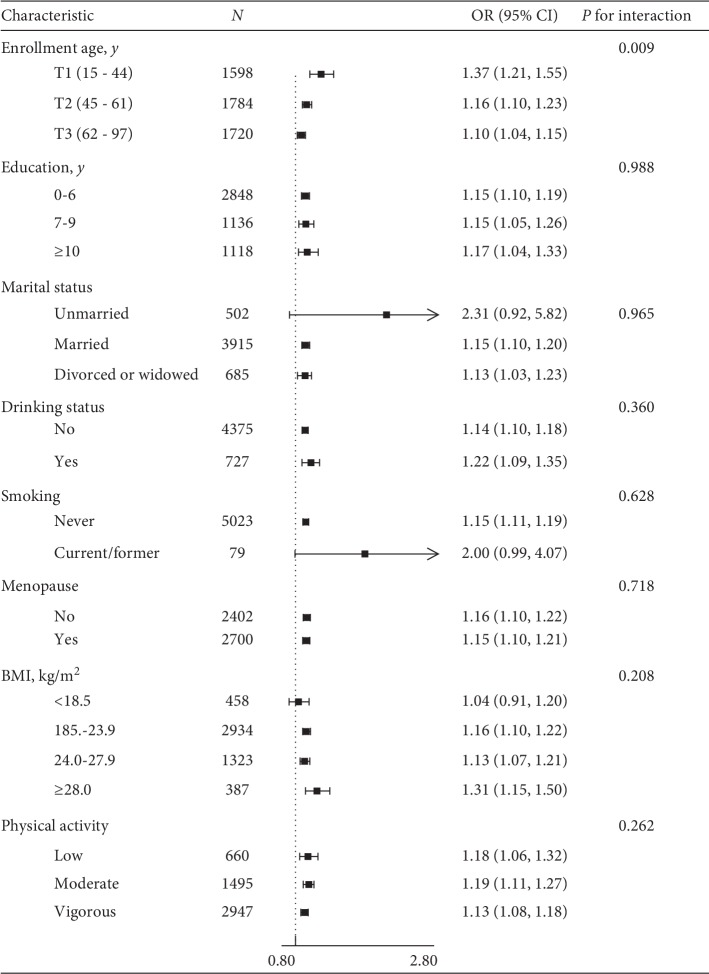
Effect size of AAM on hypertension in each subgroup. Adjusted, if not stratified, for enrollment age, residence, education, marital status, smoking, drinking, menopause, antihypertensive medication, physical activity, BMI, WC, BFP, VAI, BMR, HR, and sleeping duration.

Further subgroup analyses were performed by several important covariables, including age, education, marital status, drinking, smoking, menopause, physical activity, and BMI.  Figure 3 shows that, in multivariable-adjusted models, AAM was independently and positively associated with hypertension in all subgroups consistently. However, except the enrollment age subgroup, there were no significant interactions in any other subgroups (*P* for interaction >0.05). ORs (95% CIs) for hypertension across AAM in three enrollment age subgroups (tertile 1 : 15–44 y, tertile 2 : 45–61 y, and tertile 3 : 62–97 y) were, respectively, 1.37 (1.21–1.55), 1.16 (1.10–1.23), and 1.10 (1.04–1.15), with a decreasing trend (*P* for interaction = 0.009).

## 4. Discussion

In our study, we found that AAM was independently and positively associated with SBP and DBP and the prevalence of hypertension. For every 1-year increase in AAM, the risk of hypertension increased by 15% in all females, while SBP and DBP increased by 0.88 and 0.80 mmHg among hypertensive females. The fully adjusted smooth curve fitting presented a linear association between AAM and hypertension. Subgroup analysis showed that stronger associations between AAM and hypertension were detected in young females.

AAM serves as a significant event in the life of female, and the timing for the first menstrual period plays an important role for health of pre- and postmenopausal females [6]. Most of previous studies indicated that AAM was inversely related to hypertension worldwide, including developed Asian regions as well as some Chinese cities [8–11]. A study of 15,807 Caucasian females indicated that females with early menarche (≤12 years) had a higher risk of hypertension (OR = 1.13, 95% CI: 1.02–1.24) [8]. This study was consistent with a study of 6,242 females (21–92-year-old) as they showed that later menarche (≥18 years) was significantly correlated with lower hypertension risk (OR = 0.71, 95% CI: 0.57–0.89) [23]. A retrospective study of 13,242 subjects conducted in Chinese cities of Beijing and Xiaogan revealed that later AAM was associated with an increased risk for self-reported hypertension [24]. A study among 7,119 females from southern China reported that females with a late AAM (≥18 years) had a 39% increased risk of hypertension [12]. However, some papers had rather weak conclusions about the association between AAM and hypertension. A previous literature review on reproductive risk factors and CVD in postmenopausal females concluded that AAM had no statistically significant association with hypertension [25]. In addition, a recent study among 6,252 Chinese females from Henan Province in central China showed that females with older AAM had a higher risk of hypertension, but the association was not statistically significant [26]. Our study contributed a further evidence that late AAM increased SBP and DBP and the risk of hypertension in Chinese female population. According to the existing results, AAM was positively associated with hypertension mainly in Chinese females. The possible reasons contributing to the discrepancy included different race backgrounds, lifestyle behaviors, and living conditions between the two study populations [27, 28].

A previous cohort study reported that obesity, smoking, low physical activity, and BP increased with the time of AAM, but they interacted very weakly. In addition, educational qualifications of “college or above” were associated with later menarche [29, 30]. Lakshman et al. [8] concluded that the association between AAM and hypertension was partly mediated by adiposity. Mueller et al. [10] investigated in a large number of Chinese females in Singapore and found that a potential AAM-smoking interaction in correlation with CVD risk. In subgroup analysis, we found that the associations between AAM and hypertension were consistent in the following subgroups: education, marital status, smoking, drinking, menopause, physical activity, and BMI, whereas a stronger association between AAM and hypertension was detected in females aged 15–44 years than those aged 45 years or above. It suggests that if a female had a later AAM, she should monitor her blood pressure in their young adulthood.

According to previous studies, AAM among females in Belgium, Hungary, Britain, USA, and Scandinavia were steadily at around 13.0 ± 0.5 years [31], while the overall Chinese level of AAM was at 12.4 years in 2012 [32]. Compared with other female populations, the average AAM (15.5 years) among females in our study was 2.5 to 3.1 years later. The menarche timing was determined by genetic, living environment factors, and physical condition [33, 34]. Our study' participants were from Jiangxi Province, an underdeveloped region in southern China, where they had a low income and poor living conditions in the past. A late menarche might be due to poor nutrition in their early life, which might subsequently result in a higher risk of hypertension in late life [35]. In addition, during the years from menarche to menopause (reproductive time), females of reproductive age were continuously in estrogen exposure. Estrogen would protect females in the reproductive stage from cardiovascular diseases [25]. In our study, there were no significant differences among average age at menopause across AAM categories (≤13, 14-15, 16-17, and ≥18 years) for 2700 postmenopausal females, which meant females with a later AAM experienced shorter reproductive time. A previous study [12] on 7,119 southwestern Chinese females reported a similar conclusion. This perhaps led to an increased risk of CVD, such as hypertension [36, 37]. Moreover, our findings revealed that females with later AAM had a larger BMI, WC, BFP, and VAI during adulthood, with the similar conclusion reported in a large population-based investigation [22]. People with high levels of these physical indicators were prone to obesity. As previously reported, overweight/obesity was one of the strongest proximal risk factors of hypertension [38, 39]. In summary, the possible explanation for our finding is that a later AAM was associated with a higher risk of hypertension, mainly including poor nutrition in early life, menstruation delay, short reproductive period, different races, and lifestyle habits.

This study has some limitations. First, as a cross-sectional survey, our study could not provide the causal relationship between AAM and hypertension. Second, participants in the present study were enrolled from Jiangxi Province in southern China; therefore, the generalizability of conclusions to other regions remains to be verified. Third, AAM and some covariables were obtained according to the self-reported questionnaire responses, which might exist a bias. Finally, although we considered influence factors as many as possible, maybe other potential confounding factors were still not be included. Our study has several strengths. It was the first time to explore the exact shape of curve between AAM and hypertension among females in a developing country. All participants were randomly recruited based on city-sex-age distribution, and the representativeness of sample was better than that based on hospital data. Moreover, the information in our study was collected by face-to-face interviews, which is more reliable than telephone interviews.

## 5. Conclusion

In conclusion, our findings demonstrated that a late menarche was associated with a high BP and risk of hypertension in southern China, especially in young females. This association was independent of demographics and other risk factors. These findings indicated that knowledge of AAM could be regarded as a preventive marker for identifying females at risk of hypertension. In addition, further longitudinal epidemiological researches are needed to evaluate the effects between AAM and hypertension.

## Figures and Tables

**Table 1:** Baseline characteristics according to age at menarche.

Characteristics	Overall	Age at menarche, y				*P* value
		≤13	14-15	16-17	≥18	
*N (%)*	5,102	869	1,749	1,578	906	—
Enrollment age, y, mean (SD)	52.6 (17.7)	50.6 (18.5)	52.6 (17.6)	53.2 (17.5)	53.2 (17.5)	0.003
Age at menarche, y, mean (SD)	15.5 (2.1)	12.6 (0.7)	14.5 (0.5)	16.4 (0.5)	18.7 (1.0)	<0.001
Menopause, *n*(%)	2700 (52.92%)	276 (31.76%)	815 (46.60%)	956 (60.58%)	653 (72.08%)	<0.001
Hypertension, *n*(%)	1,495 (29.3%)	204 (23.5%)	427 (24.4%)	538 (34.1%)	326 (36.0%)	<0.001
Antihypertensive medication, *n*(%)	375 (7.4%)	58 (6.7%)	101 (5.8%)	141 (8.9%)	75 (8.3%)	0.003
Residence (urban), *n*(%)	2528 (49.6%)	430 (49.5%)	871 (49.8%)	803 (50.9%)	424 (46.8%)	0.271

*Education, y, n*(%)						0.370
0–6	2848 (55.8%)	464 (53.4%)	961 (54.9%)	905 (57.4%)	518 (57.2%)	
7–9	1136 (22.3%)	196 (22.6%)	394 (22.5%)	352 (22.3%)	194 (21.4%)	
≥10	1118 (21.9%)	209 (24.1%)	394 (22.5%)	321 (20.3%)	194 (21.4%)	

*Marital status, n*(%)						0.033
Unmarried	502 (9.8%)	112 (12.9%)	175 (10.0%)	138 (8.8%)	77 (8.5%)	
Married	3915 (76.7%)	648 (74.6%)	1332 (76.2%)	1231 (78.0%)	704 (77.7%)	
Divorced or widowed	685 (13.5%)	109 (12.5%)	242 (13.8%)	209 (13.2%)	125 (13.8%)	

*Smoking, n*(%)						0.860
Never	5023 (98.4%)	854 (98.3%)	1724 (98.5%)	1550 (98.2%)	895 (98.8%)	
Current	70 (1.4%)	13 (1.5%)	22 (1.3%)	26 (1.7%)	9 (1.0%)	
Former	9 (0.2%)	2 (0.2%)	3 (0.2%)	2 (0.1%)	2 (0.2%)	

*Drinking, n*(%)	727 (14.3%)	119 (13.7%)	245 (14.0%)	231 (14.6%)	132 (14.6%)	0.902
*Physical activity, n*(%)						0.008
Low	660 (12.9%)	117 (13.5%)	215 (12.3%)	180 (11.4%)	148 (16.3%)	
Moderate	1495 (29.3%)	265 (30.5%)	534 (30.5%)	457 (29.0%)	239 (26.4%)	
Vigorous	2947 (57.8%)	487 (56.0%)	1000 (57.2%)	941 (59.6%)	519 (57.3%)	
BMI, kg/m^2^, mean (SD)	22.8 (3.6)	22.4 (3.3)	22.84 (3.6)	23.0 (3.7)	22.9 (3.6)	<0.001
WC, cm, mean (SD)	77.8 (9.6)	76.8 (9.6)	77.9 (9.3)	78.2 (9.6)	78.0 (9.8)	0.005
BFP, %, mean (SD)	30.2 (7.7)	29.5 (7.6)	30.1 (8.0)	30.5 (7.4)	30.3 (7.6)	0.035
VAI, mean (SD)	6.3 (3.7)	6.0 (3.4)	6.3 (3.6)	6.5 (3.8)	6.4 (4.0)	0.025
BMR, kcal/day, mean (SD)	1167.9 (312.0)	1153.9 (266.8)	1164.2 (296.3)	1178.9 (366.8)	1169.6 (275.5)	0.265
HR, mean (SD)	78.8 (11.1)	78.4 (10.4)	78.7 (11.0)	78.9 (11.3)	79.1 (11.4)	0.543
SBP, mmHg, mean (SD)	124.7 (21.6)	121.6 (19.1)	122.6 (20.4)	126.5 (22.5)	128.5 (23.5)	<0.001
DBP, mmHg, mean (SD)	72.9 (11.5)	71.2 (8.9)	72.14 (11.0)	73.8 (12.33)	74.7 (12.6)	<0.001
Sleeping duration, h, mean (SD)	7.3 (1.6)	7.3 (1.6)	7.2 (1.6)	7.2 (1.7)	7.2 (1.6)	0.884

BMI = body mass index; BMR = basal metabolism rate; WC = waist circulation; BFP = body fat percentage; VAI = visceral adiposity index; SBP = systolic blood pressure; DBP = diastolic blood pressure; HR = heart rate.

**Table 2:** Analysis of AAM and hypertension.

AAM	*N*	Hypertension OR (95% CI), *P* value			
		Cases	Crude	Model 1	Model 2
Continuous variable	5,102	1,495	1.13 (1.10, 1.16), <0.001	1.14 (1.10, 1.17), <0.001	1.15 (1.11, 1.19), <0.001

*Categorical variables*					
≤13 y	869	204	1.00	1.00	1.00
14-15 y	1,749	427	1.05 (0.87, 1.27), 0.597	0.97 (0.78, 1.20), 0.765	0.95 (0.76, 1.19), 0.681
16-17 y	1,578	538	1.69 (1.40, 2.04), <0.001	1.65 (1.43, 2.02), <0.001	1.68 (1.34, 2.11), <0.001
≥18 y	906	326	1.83 (1.49, 2.25), <0.001	1.89 (1.50, 2.40), <0.001	2.01 (1.56, 2.58), <0.001
*P* for trend	—	—	<0.001	<0.001	<0.001
≤15 y	2,618	631			
16-17 y	1,578	538	1.63 (1.42, 1.87), <0.001	1.68 (1.44, 1.97), <0.001	1.74 (1.48, 2.05), <0.001
≥18 y	906	326	1.77 (1.50, 2.08), <0.001	1.94 (1.61, 2.33), <0.001	2.08 (1.71, 2.53), <0.001
*P* for trend	—	—	<0.001	<0.001	<0.001

Model 1 included enrollment age, residence, education, and marital status for adjustment. Model 2 included enrollment age, residence, education, marital status, smoking, drinking, menopause, antihypertensive medication, physical activity, BMI, WC, BFP, VAI, BMR, HR, and sleeping duration. BMI = body mass index; BMR = basal metabolism rate; WC = waist circulation; BFP = body fat percentage; VAI = visceral adiposity index; HR = heart rate.

**Table 3:** Analysis of AAM and SBP, and DBP in females with hypertension.

AAM, y	*N*	Mean (SD)	*β* (95% CI), *P* value		
			Crude	Model 1	Model 2
*SBP*					
Continuous variable, y	1,495	146.9 (24.3)	0.86 (0.28, 1.45), 0.004	0.98 (0.40, 1.56), 0.001	0.88 (0.29, 1.46), 0.003
Categorical variables					
≤15 y	631	144.5 (25.6)	0	0	0
16-17 y	538	147.8 (23.0)	3.32 (0.54, 6.10), 0.020	3.72 (0.95, 6.49), 0.009	3.50 (0.72, 6.27), 0.014
≥18 y	326	150.2 (23.3)	5.67 (2.44, 8.90), 0.001	6.41 (3.20, 9.63), <0.001	5.84 (2.62, 9.05), <0.001
*P* for trend	—	—	<0.001	<0.001	<0.001

*DBP*					
Continuous variable	1,495	80.6 (14.4)	0.86 (0.52, 1.21), <0.001	0.88 (0.54, 1.22), <0.001	0.80 (0.47, 1.13), <0.001
Categorical variables					
≤15 y	631	78.2 (14.7)	0	0	0
16-17 y	538	82.3 (13.9)	4.07 (2.43, 5.71), <0.001	4.09 (2.48, 5.71), <0.001	3.35 (1.81, 4.88), <0.001
≥18 y	326	82.7 (13.9)	4.50 (2.60, 6.41), <0.001	4.54 (2.66, 6.42), <0.001	4.34 (2.53, 6.16), <0.001
*P* for trend	—	—	<0.001	<0.001	<0.001

Model 1 adjusted for enrollment age, residence, education, and marital status for adjustment. Model 2 adjusted for enrollment age, residence, education, marital status, smoking, drinking, menopause, antihypertensive medication, physical activity, BMI, WC, BFP, VAI, BMR, HR, and sleeping duration. BMI = body mass index; BMR = basal metabolism rate; WC = waist circulation; BFP = body fat percentage; VAI = visceral adiposity index; HR = heart rate.

## Data Availability

The data used to support the findings of this study are available from the corresponding author upon request.
